# Organized chronic subdural hematoma with cognitive impairment: A case report and literature review

**DOI:** 10.1097/MD.0000000000041260

**Published:** 2025-01-31

**Authors:** Sen He, Fang Xue, Jing Li, Jianqiang Hao, Wenyan Zhang, Fei Xie

**Affiliations:** a Department of Neurosurgery, The Ziyang Central Hospital, Ziyang, Sichuan, China; b Department of Pathology, The Ziyang Central Hospital, Ziyang, Sichuan, China.

**Keywords:** case report, chronic subdural hematoma, cognitive impairment, diagnosis, neuroimaging, organic chronic subdural hematoma

## Abstract

**Rationale:**

Organic chronic subdural hematoma is extremely rare in clinical practice, with unclear etiology and pathogenesis. Its clinical manifestations and treatment approaches are diverse, making diagnosis challenging and prone to misdiagnosis or mistreatment, adversely affecting patient care and quality of life.

**Patient concerns:**

The 58-year-old male patient exhibited cognitive impairment, characterized by memory deficits and delayed responses, over 1 month in the absence of notable medical comorbidities.

**Diagnoses:**

Initial neurological assessment upon admission showed cognitive deficits, with a Mini-Mental State Examination score of 18 and a Montreal Cognitive Assessment Scale score of 22. Imaging with a computed tomography scan revealed a subdural mass with low density. The preoperative diagnosis indicated a chronic subdural hematoma (may combined with intracranial hypertension) located at the apex of the right frontotemporal region, potentially with septation.

**Interventions:**

A bone flap craniotomy was proceeded under microscopic guidance for lesion resection. Postoperatively, the patient received targeted interventions, including fluid replacement, to promote brain tissue recovery and functional rehabilitation.

**Outcomes:**

After treatment, the patient demonstrated improvement and was discharged from the hospital. Over the 1-year postoperative period, he reported mild recent memory decline but remained asymptomatic, continued his usual activities, and demonstrated improved cognitive function, as evidenced by Mini-Mental State Examination and Montreal Cognitive Assessment Scale scores of 28 and 29, respectively, along with normal muscle strength in all limbs.

**Lessons:**

Patients with mild or no significant symptoms, such as headaches and dizziness, are advised to undergo regular imaging follow-ups. Surgical intervention is recommended for patients presenting with intracranial hypertension and neurological impairment, with bone flap craniotomy and lesion resection under microscopic guidance being the preferred approach. It is imperative to conduct regular postoperative monitoring to promptly detect potential complications, such as hematoma recurrence.

## 1. Introduction

Chronic subdural hematoma is a prevalent neurosurgical condition that predominantly affects middle-aged and elderly individuals.^[1]^ Chronic subdural hematoma increases slowly, usually symptomsizes after 2 to 3 weeks due to direct brain pressure and increased intracranial pressure.^[1]^ Chronic subdural hematoma is easy to be misdiagnosed as intracranial tumor, intracranial ischemia or hemorrhagic acute cerebrovascular disease before diagnosis by imaging. Chronic subdural hematoma typically occurs between the dura and arachnoid membranes, comprising approximately 10% of intracranial hematomas.^[2]^ In contrast, organized chronic subdural hematoma (OCSDH) is a rare clinical phenomenon with limited documented reports.^[3]^ OCSDH is characterized by clots and calcifications, manifesting as multiple lobulations with hematomas of mixed density on brain CT scans. Unlike non-organized chronic subdural hematoma cases, some OCSDH cases often necessitate urgent burr-hole drainage due to the rapidly progressive neurological symptoms; but most instances of OCSDH do not demonstrate worsening conditions and generally do not require immediate surgical intervention.^[4]^ However, distinguishing between these 2 conditions can be very challenging due to the elevated CT values associated with OCSDH.

Although the most effective treatment strategy for OCSDH remains uncertain,^[5]^ OCSDH is mainly treated with surgical strategy to minimize the less risk of the patients. A large craniotomy is recommended as a radical solution despite the risks associated with postoperative hemorrhagic complications and recurrence. As reported, a large craniotomy for OSDH may possibly carry a high rate of mortality and morbidity, especially in those patients with old age. Seizures, tension pneumocephalus and recurring subdural hemorrhage are the commonly seen complications in these patients after surgery.^[5]^

Given the atypical early symptoms, insidious onset, and slow progression of most OCSDH cases, misdiagnoses are prevalent among patients.^[6]^ The challenges in fully understanding the pathogenesis of OCSDH and establishing standardized treatment protocols persist in the field. This paper presents a detailed case study of individuals suffering from cognitive deficits caused by OCSDH and comprehensively reviews the current literature. By investigating clinical presentations, diagnostic approaches, treatment options, and outcomes related to OCSDH, this study aims to advance the comprehension and management of this uncommon subtype of subdural hematoma.

## 2. Case report

The work was approved by the Ethical Committee of Ziyang Central Hospital (2024-Ethical Review-No.199). The patient has given his written informed consent for this study and the publication of his related data.

### 2.1. Patient complaint

The 58-year-old male patient was admitted on July 10, 2023, with a 1-month history of cognitive impairment characterized by memory deficits and delayed responses. He had no prior medical history of cerebral hemorrhage, hypertension, diabetes, Alzheimer disease, or head trauma. He reported headache for sometimes.

### 2.2. Examinations upon admission

**Figure 1.**
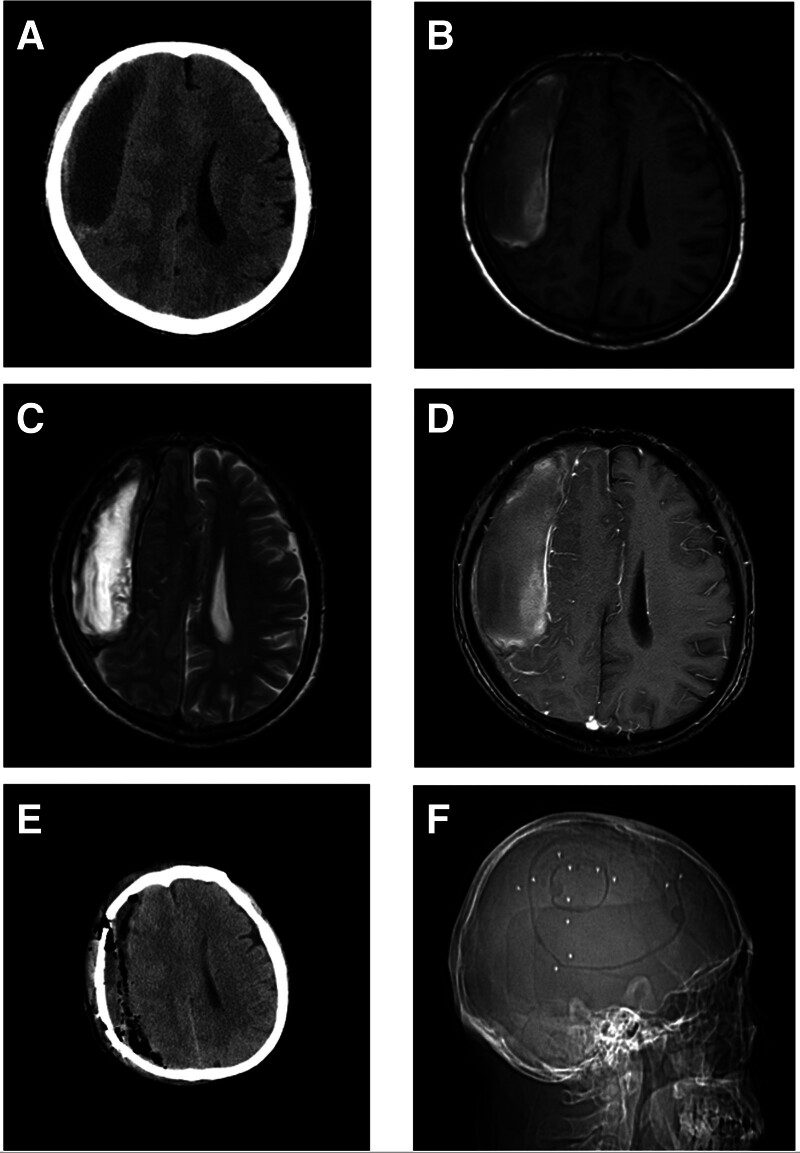
Imaging examination results of the patient. (A) The preoperative CT scan revealed a low-density subdural mass located in the right frontal, temporal, parietal, and occipital regions, causing a midline shift towards the contralateral side. (B) The preoperative T1 sequence revealed mixed signal shadows, predominantly exhibiting high signal intensity in the subdural region of the right frontal, temporal, parietal, and occipital lobes. (C) The preoperative T2 sequence revealed hypointense shadows between the lesion and the cerebral cortex, with no apparent edema signal surrounding the lesion. (D) No significant enhancement was detected within the lesion on the preoperative MR enhancement scan, with linear enhancement shadows noted along the lesion’s periphery bordering the cerebral cortex. (E) The postoperative CT scan indicated the clearance of the right subdural hematoma and a reduction in midline structural deviation. (F) The postoperative CT localization film revealed both a small bone window craniotomy shadow and a large bone flap craniotomy shadow.

Upon admission, his vital signs were stable, showing a blood pressure of 135/74 mm Hg, a heart rate of 88 beats per minute, and a Glasgow Coma Scale score of 15. The neurological assessment revealed bilateral pupils measuring 4 mm with intact light reflexes and cognitive deficits encompassing memory, computational ability, abstract reasoning, and language skills. The patient scored 18 on the Mini-Mental State Examination and 22 on the Montreal Cognitive Assessment Scale. His limb muscle tone was normal, with left-sided muscle strength graded at level 4 and right-sided strength at level 5. Laboratory investigations, including blood tests, coagulation studies, and assessments of liver and kidney function, as well as stool and urine analyses, all fell within normal ranges. A CT scan revealed a low-density subdural mass located in the right frontal, temporal, parietal, and occipital regions, with high-density areas at the lesion’s periphery and evidence of midline shift. MRI scans exhibited mixed signal intensities in the subdural regions, with low signal regions adjacent to the cerebral cortex but without surrounding edema. Contrast-enhanced MRI did not show significant enhancement within the lesion, although linear enhancement was observed at the lesion’s border near the cerebral cortex (Fig. 1). The patient had symptoms of headache, and intracranial hypertension was considered in combination with imaging examination (local brain tissue compression and midline structural shift). Based on previous researched,^[1–4]^ intracranial hypertension may be caused by OCSDH.

### 2.3. Diagnosis and treatment

**Figure 2.**
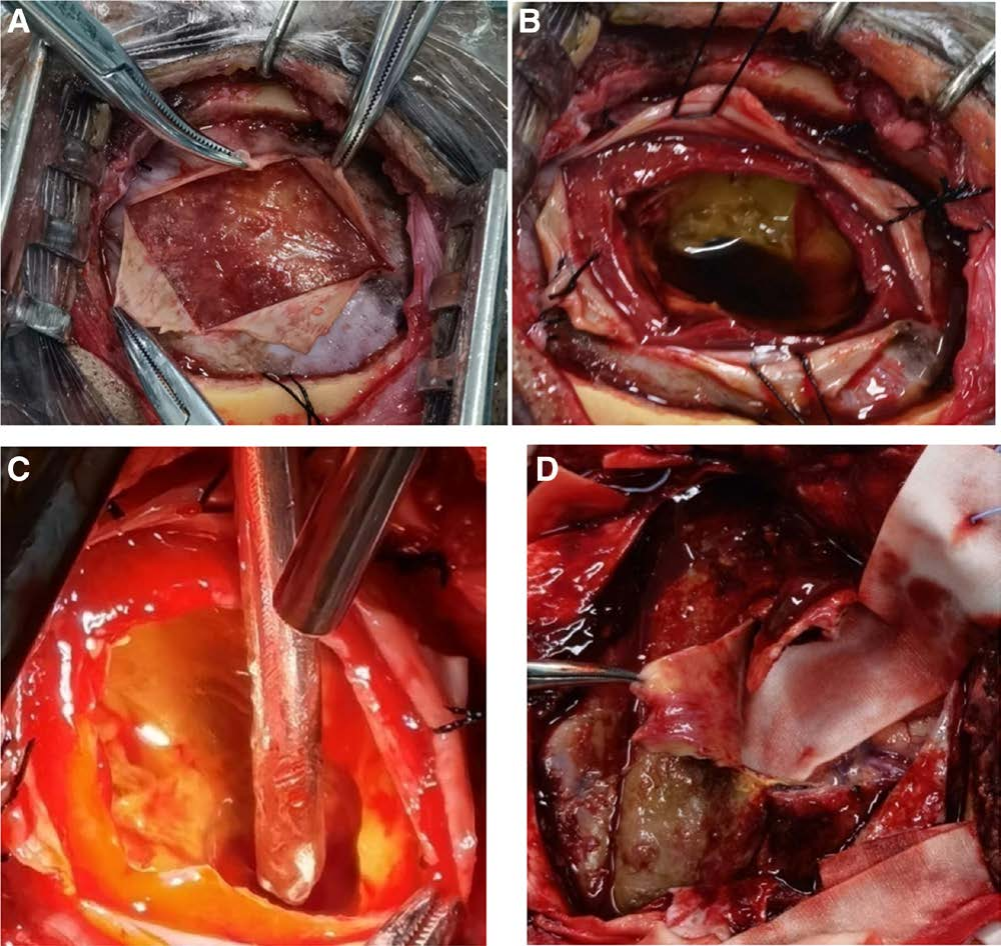
Intraoperative assessment of patient’s condition. (A) During a small bone window craniotomy, an incision of the dura mater exposed a hard, light-yellow subcapsular layer beneath it, clearly demarcated from the dura mater. (B) Following the capsule incision, a layer measuring around 2 mm to 4 mm thick was identified, which held a minor quantity of aged bloody fluid and blood clots. (C) After removing bloody fluids and clots, a substantial quantity of solid yellow cheese-like substances was visible, accompanied by a limited blood supply. Some of these could be extracted using an aspirator. (D) After a craniotomy involving a large bone flap, the visceral capsule was observed to measure approximately 3 mm in thickness, appear gray-white, and exhibit a notable proliferation of capillaries on the cortical surface.

The preoperative diagnosis indicated a chronic subdural hematoma (may combined with intracranial hypertension) located at the apex of the right frontotemporal region, potentially with septation. Figure 2 shows the patient’s intraoperative condition. A craniotomy with a small bone window on the right temporal region was performed for lesion removal using neuroendoscopy. No fluid was visualized upon the dura mater incision. Subsequent enlargement of the dural incision revealed a 2- to 4-mm thick, tough, hard gray-white, and light-yellow capsule with clear demarcation from the dura mater and loose adhesions. Upon capsule incision, a small quantity of aged brown bloody fluid and blood clots were encountered, followed by a substantial amount of yellow, cheese-like solid material after clearing the bloody components. The vascularity was limited, with some sections being soft and friable, removable via negative pressure suction, while others were resilient and necessitated block-wise extraction. Despite most lesions being excised via neuroendoscopy, the instruments could not access the lesion’s periphery, hindering complete removal and hemostasis. The inability to effectively separate and excise the capsule wall at the lesion’s periphery resulted in insufficient brain tissue removal. With the patient’s consent, the original incision was enlarged to perform a bone flap craniotomy under microscopic guidance for lesion resection. Intraoperatively, it was observed that the lesion’s wall envelope folded back at the periphery, forming a visceral envelope on the arachnoid membrane surface. The demarcation between the wall envelope and dura mater was clear, while that between the visceral envelope and arachnoid membrane was indistinct. Notably, significant capillary proliferation was evident in the cortical cystic wall. Complete removal of the capsule and its contents was carried out for histopathological analysis.

### 2.4. Postoperative follow-up

Postoperatively, the patient received targeted interventions, including fluid replacement, to promote brain tissue recovery and functional rehabilitation. After treatment, the patient demonstrated improvement and was discharged from the hospital.

**Figure 3.**
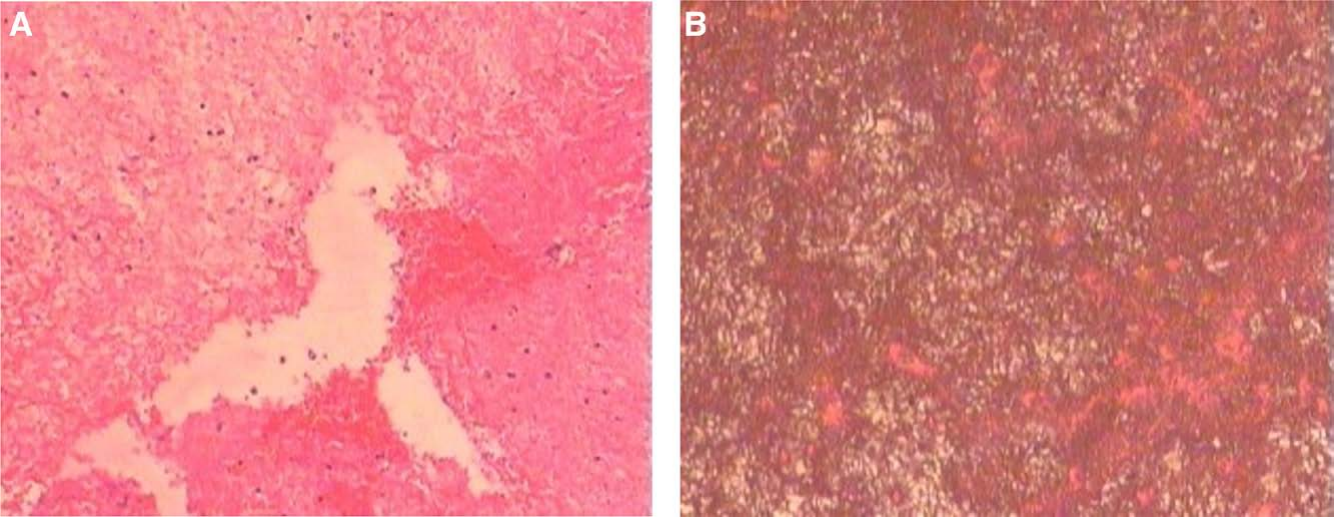
Postoperative pathological assessment of the patient. (A) Through HE staining, the capsule wall exhibited fibrous tissue, blood clots, inflammatory cells, and hemosiderin deposition (×100). (B) Through HE staining, the capsule contents exhibited hyperplastic and hyaline fibrous connective tissue, blood clots, inflammatory necrosis, exudates, and focal foam-like tissue cell aggregation (×100).

Postoperative pathological examination (Fig. 3) of the capsule wall revealed fibrous cystic tissue with blood clots, inflammatory cells, and hemosiderin deposition. The capsule contents exhibited hyperplastic and hyaline fibrous connective tissue, blood clots, inflammatory necrosis, exudates, and localized aggregation of foam-like tissue cells.

**Figure 4.**
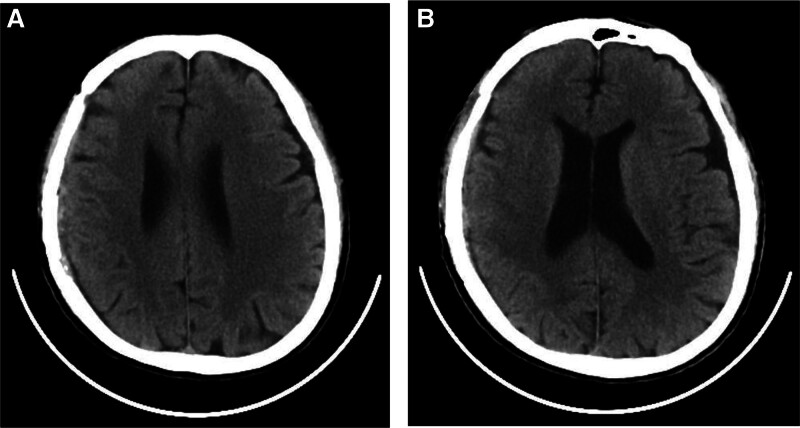
The results of the head CT scan of the patient 1-year post-surgery. (A) The low-density subdural mass in the right frontal, temporal, parietal, and occipital regions was resolved. (B) The centerline structure was centered.

The patient was required to come back to hospital and do follow-up examinations every 3 months. He recovered well and did not reported any severe complications. At 1-year postoperative follow-up, the patient reported a mild recent decline in memory, with no concomitant symptoms such as headaches or dizziness, while maintaining normal daily activities. Cognitive functions, including executive function, abstract reasoning, and language skills, were assessed within normal limits, with scores of 28 on the Mini-Mental State Examination and 29 on the Montreal Cognitive Assessment Scale. Additionally, the patient demonstrated normal muscle tone and muscle strength graded at level 5 in all limbs. A subsequent head CT scan (Fig. 4) revealed a significant decrease in the low-density mass located beneath the dura mater in the right frontal, temporal, parietal, and occipital regions, with a well-centered midline structure. Since then, the doctor proceeded telephone follow-up with the patient every 2 months. Until November 30, 2024, the patient reported of good health vie telephone contact.

## 3. Discussion

OCSDH is a rare, benign lesion characterized by a cystic solid formation located between the dura mater and the arachnoid membrane. Despite its low incidence in clinical practice, OCSDH typically exhibits an indolent onset and a prolonged course.^[7]^ The precise etiology and pathogenesis of this condition remain unclear; nonetheless, it is thought to be precipitated by minor trauma or other factors resulting in intracranial hemorrhage and subsequent hematoma accumulation in the subdural space. In cases of brain atrophy, the increased cranial cavity volume compensates for any space-occupying lesions within the skull, thus delaying the onset of clinical symptoms.^[7]^ The hematoma solidifies before liquefaction, leading to the continuous deposition of fibrous material, primarily attributed to non-degrading hemosiderin, culminating in capsule formation and hematoma organization. In contrast to typical mechanization processes, the pathological tissue in OCSDH lacks cellular components, prompting some researchers to classify it as “sediment type chronic subdural hematoma” or “rigid type chronic subdural hematoma”.^[8]^ Our documented case did not demonstrate granulation tissue elements in the cystic contents, further supporting this classification. OCSDH predominantly affects middle-aged and elderly individuals, presenting with a range of nonspecific symptoms such as dizziness, headache, epilepsy, hemiplegia, sensory disturbances, cognitive impairment, and urinary incontinence. Due to its insidious onset and protracted progression, OCSDH is susceptible to misdiagnosis or oversight without appropriate neuroimaging. Our patient was 58 years old, which is in compliance with previous studies.^[6–8]^ The patient also reported of headache, which may be caused by enlarged hematoma during the development of OCSDH. This patient exhibited symptoms of “poor memory and delayed reaction for 1 month.” These symptoms cannot be found easily in clinical practice, and will easily misdiagnosed as neurosis or senile dementia. The asymptomatic characteristics of OCSDH highlight the risk of underdiagnosis in the absence of imaging studies, and the important value of imaging tools in diagnosing OCSDH patients.

CT and MRI are common techniques for diagnosing neurological diseases, including OCSDH. On CT, OCSDH appears as well-defined heterogeneous masses located beneath the dura mater and external to the cerebral cortex.^[9]^ These masses display hyperdense peripheries and hypodense interiors, which looked like a density area of crescent, half-moon or double convex spherical lens under the inner plate of the skull. MRI reveals OCSDH as abnormal signal characteristics between the dura mater and cerebral cortex, with hypointense margins on both T1- and T2-weighted sequences and varying internal signal features.^[10]^ Contrast-enhanced MRI scans show accentuated margins without internal enhancement. Our case aligns with these imaging findings. While CT and MRI are essential for diagnosis, differential diagnosis, and treatment planning of OCSDH, they are insufficient for preoperative diagnosis. Clinically, distinguishing OCSDH from conditions like meningioma, lymphoma, glioma, metastasis, intracranial abscess, and parasitic diseases, especially from chronic subdural hematoma, is crucial. OCSDH typically presents a prolonged clinical course and thicker capsule, with calcification aiding differentiation.^[11]^ However, in cases where chronic subdural hematoma shows thick capsules, incomplete liquefaction, and septation, intraoperative observations, and histopathology are required for clarification. Surgical exploration typically reveals chronic subdural hematoma as a mixture of bloody fluid and clots, while OCSDH appears as a solid lesion with some fluid content. Histopathological analysis is vital for ruling out neoplastic conditions such as meningioma and lymphoma to ensure an accurate diagnosis.^[12]^ Therefore, imaging tools and histopathological analysis are necessary for the diagnosis of OCSDH, which are also used in the current case report.

The management of chronic subdural hematoma lacks established guidelines or consensus, with a focus on patients with small lesions, no significant mass effect, and clinical discomfort. Treatment may involve medication, close monitoring, and regular imaging. Common drugs used include atorvastatin,^[13]^ glucocorticoids,^[14]^ or a combination,^[15]^ but caution is warranted due to potential adverse reactions and varied patient responses. A previous report by Akaishi et al has reported successful cases of avoiding surgical intervention for chronic subdural hematoma through vigilant observation and follow-up.^[4]^ To accurately confirm the patient’s condition, the doctors should comprehensively consider the patients’ clinical symptoms, imaging examinations and close monitoring.

Surgical intervention is essential for patients with significant symptoms or large lesions causing noticeable space-occupying effects. While drilling and drainage surgery are the preferred approach for patients with chronic subdural hematoma,^[16–19]^ it is ineffective for OCSDH due to the lesion’s solid nature with minimal liquid components. This method only addresses draining the liquid component, failing to alleviate the mass effect of the lesion. Moreover, retaining the capsule during surgery results in poor brain tissue compliance, inadequate postoperative brain expansion, and an increased risk of recurrence. Therefore, for OCSDH cases, performing bone flap craniotomy under a microscope for lesion resection is recommended.^[20,21]^ Kameda et al reported a very special and complex case that Epstein–Barr virus-positive diffuse large B-cell lymphomathe developed in the OCSDH; the membranes of the subdural hematoma in the patient reported were all fibrous; this patient was cured by surgical intervention, too.^[20]^ During craniotomy, the bone flap should be sufficiently large to reach or nearly reach the capsule’s edge, enabling complete capsule removal and precise hemostasis under a microscope. This meticulous approach helps prevent uncontrolled bleeding, reduces surgical complexity, and minimizes postoperative complications. In cases where a small bone window craniotomy is performed, access to the lesion’s edge may be compromised, necessitating an expanded bone window for comprehensive lesion removal. Caution must be exercised when dealing with large lesions near the midline or sagittal sinus to avoid sinus damage and intraoperative bleeding. Gradual decompression is crucial during the evacuation of lesion contents to reduce the risk of ischemia–reperfusion injury and secondary bleeding. Delicate separation is essential when removing the visceral capsule to prevent injury to the arachnoid membrane, cerebral cortex, and vital blood vessels located beneath the capsule. In addition, the craniotomy should be proceeded by experience surgeons to ensure its optimal intervention effect.

Baek et al^[22]^ highlighted the importance of complete resection of the capsule during surgery to prevent residual edge bleeding, a significant factor in the occurrence and recurrence of acute subdural hematomas, chronic subdural hematoma, and postoperative brain swelling. Total capsulectomy was performed in the current case due to the weak adherence between the patient’s capsule and cortex. The relationship between the visceral capsule and the risk of epilepsy remains uncertain. Therefore, if separating the visceral capsule from the arachnoid membrane presents challenges, it is advisable not to forcibly detach it to avoid harm to the arachnoid membrane, cerebral cortex, and major blood vessels, thus reducing the likelihood of postoperative complications like fluid accumulation, epilepsy, and impaired brain function. When considering partial resection of the visceral capsule layer, it is recommended to refrain from extensive “window opening” procedures to prevent local brain herniation caused by compression and entrapment in the weakened area following postsurgical brain tissue relaxation.^[23]^ The detailed surgical treatment should be considered and proceeded based on the clinical situation and status of the patients, and the optimal choice should be chosen based on comprehensive surgical indications.

Neuroendoscopic surgery is a vital technique for managing chronic subdural hematoma and recurrent chronic subdural hematoma cases characterized by complex features such as septations, encapsulation, and hematoma organization.^[19,24]^ Its advantages include minimal invasiveness, improved visualization, and shorter surgical duration. This method enables the precise removal of components of chronic subdural hematoma like membranes, septae, and hematomas, ensuring thorough exposure and optimal visualization while minimizing surgical risks and reducing postoperative complications such as recurrence and rebleeding.^[19,25]^ Wu et al find that neuroendoscopy-assisted surgical treatment shortens the postoperative drainage time, and reduces the recurrence rate of CSDH than conventional burr-hole evacuation, with a comparable mortality and postoperative morbidity rate.^[25]^ Neuroendoscopy has proven effective in evacuating organized hematomas and tissues in outer chronic subdural hematoma cases.^[26]^ However, challenges arise when the lesion presents as a widespread “flying saucer” or “fried egg” configuration, creating visual and operational blind spots that impede complete resection and hemostasis. In such cases, enlarging the bone window is necessary to achieve complete lesion clearance.

Middle meningeal artery embolization is a surgical procedure that reduces dural blood supply, inhibits neovascularization, suppresses inflammatory mediator release, and prevents hematoma formation and recurrence to achieve therapeutic goals.^[27]^ While middle meningeal artery embolization has demonstrated efficacy in chronic subdural hematoma, its initial onset is not prevented.^[28]^ In OCSDH, characterized by a lack of liquid components within the lesion, the clinical effectiveness of inhibiting neovascularization to reduce exudation or hemorrhaging is limited. Additionally, the blood supply to the lesion originates not only from the dura mater but also from the lesion’s visceral layer, raising questions about the standalone efficacy of middle meningeal artery embolization in treating OCSDH. Nonetheless, some studies suggest that middle meningeal artery embolization can reduce perioperative bleeding risk and postoperative recurrence during craniotomy.^[6,29]^ It is important to note that middle meningeal artery embolization targets the middle meningeal artery specifically and does not directly address the mass effect of the lesion. The therapeutic effects of middle meningeal artery embolization are typically delayed post-surgery and may be restricted in patients with acute and severe clinical presentations. Furthermore, there is a lack of consensus regarding target vessel selection, protection of anastomotic branches, and the choice of embolization materials for middle meningeal artery embolization, highlighting the need for further research. It is crucial to remain vigilant against potential complications such as visual impairment, hemorrhage, infarction, allergies to contrast agents, and catheter-related issues. In this study, the patient recovered smoothly after complete lesion clearance without complications, which stated that surgical treatment is effective for OCSDH.

The clinical prognosis of chronic subdural hematoma is generally favorable. However, factors such as advanced age, significant brain atrophy, prolonged disease duration, and substantial compression of local brain tissue can hinder postoperative brain recovery and increase the risk of hematoma recurrence. Surgical separation of the outer membrane during the procedure may lead to cerebral cortex damage or invasion, potentially triggering epilepsy. Moreover, there is a risk of postoperative bleeding and hematoma formation outside the resected area. Hence, meticulous postoperative imaging surveillance and regular follow-up are essential. The current case reported no severe complications vie every 3 months’ follow-up within 1 year. The close follow-up with the patient provided a good monitoring on the patient.

In conclusion, OCSDH is a rare condition in clinical practice, characterized by a gradual onset, prolonged duration, and unusual clinical presentations, often leading to misdiagnosis as chronic subdural hematoma. Diagnostic imaging through head CT and MR scans plays a pivotal role in identifying and managing this condition. Patients with mild or no significant symptoms, such as headaches and dizziness, are advised to undergo regular imaging follow-ups. Before considering pharmacological interventions, a comprehensive assessment of the benefits and potential adverse reactions is essential. Surgical intervention is recommended for patients presenting with intracranial hypertension and neurological impairment, with bone flap craniotomy and lesion resection under microscopic guidance being the preferred approach. In cases where the diagnosis of OCSDH remains uncertain before surgery, initial drilling and exploration procedures may be conducted. A preliminary diagnosis of OCSDH can be made if a thick-walled capsule containing yellow, cheese-like solid material and a small amount of bloody fluid is identified beneath the dura mater. Subsequently, enlarging the incision and performing lesion resection under microscopic visualization is warranted. For smaller lesions, neuroendoscopic resection is a viable alternative. During surgical intervention, thoroughly removing the capsule and its contents is crucial. However, if the visceral capsule is tightly adhered to the arachnoid membrane, caution should be exercised to prevent damage to critical structures. Despite the generally favorable surgical outcomes, it is imperative to conduct regular postoperative monitoring to promptly detect potential complications, such as hematoma recurrence.

## Author contributions

**Conceptualization:** Jing Li.

**Data curation:** Sen He, Fang Xue, Jing Li, Jianqiang Hao.

**Formal analysis:** Sen He.

**Funding acquisition:** Fei Xie.

**Investigation:** Wenyan Zhang.

**Methodology:** Sen He, Fang Xue, Jianqiang Hao.

**Project administration:** Sen He, Fei Xie.

**Writing – original draft:** Sen He.

**Writing – review & editing:** Sen He, Fei Xie.
